# 

**Published:** 1892-09

**Authors:** 


### TABLE OF CONTENTS.

ORIGINAL COMMUNICATIONS. PAGE

Demonstration of the Reticulum in Dentine with Low Powers of the Microscope. By C. Heitzmann, M.D. . . . . . . . . . . . . . . . . 629

Crown- and Bridge-Work. (*Continued.*) By Dr. C. M. Richmond, New York . . . . . . . . . . . . . . . . . . . . . . . . . 634

Primary and Storage Batteries for Dental Purposes. By Jere. E. Stanton, M.D., D.M.D., Boston, Mass. . . . . . . . . . . . . . . . . . . . . . 637

Dental Education. By W. Xavier Sudduth, A.M., M.D., D.D.S. . . . 645

Silica: Its Curative Action in the Treatment of Alveolar Abscess. By Charles H. Taft, A.B., D.M.D. . . . . . . . . . . . . . . . . . . 653

Argenti Nitras as a Remedy in the Treatment of Pyorrhœa. By Dr. E. H. Stebbins, Shelburne Falls, Mass. . . . . . . . . . . . . . . . . . . . 659

REPORTS OF SOCIETY MEETINGS.

New York Odontological Society . . . . . . . . . . . . . . . . . 663

American Dental Association . . . . . . . . . . . . . . . . . . . 671

American Academy of Dental Science . . . . . . . . . . . . . . . 678

National Association of Dental Faculties . . . . . . . . . . . . 688

National Association of Dental Examiners . . . . . . . . . . . . 692

EDITORIAL.

The Annual Meetings at Niagara . . . . . . . . . . . . . . . . . 695

The World's Columbian Dental Congress . . . . . . . . . . . . . 697

Women's Dental Association . . . . . . . . . . . . . . . . . . . 698

American College of Dental Surgery, Chicago . . . . . . . . . . 698

BIBLIOGRAPHY . . . . . . . . . . . . . . . . . . . . . . . . . . 699

DOMESTIC CORRESPONDENCE.

Dr. Barrett's Statement . . . . . . . . . . . . . . . . . . . . . 700

The Women's Dental Association of the United States . . . . . . 702

CURRENT NEWS.

New Jersey Examinations . . . . . . . . . . . . . . . . . . . . . 704

Indiana State Dental Association . . . . . . . . . . . . . . . . 704

Connecticut Valley Dental Society . . . . . . . . . . . . . . . . 704

Annual Meeting of the Alumni Association of the New York College of Dentistry . . . . . . . . . . . . . . . . . . . . . . . . . . . . 705

SELECTIONS.

Dentists in the Army and Navy . . . . . . . . . . . . . . . . . . 705

